# Identification and validation of lipid metabolism-related key genes as novel biomarkers in acute myocardial infarction and pan-cancer analysis

**DOI:** 10.18632/aging.205860

**Published:** 2024-05-23

**Authors:** Hao Xiao, Xiaolei Cui, Liang Liu, Baopu Lv, Rui Zhang, Tuokang Zheng, Dongqi Yao, Hengbo Gao, Xinshun Gu, Yi Li, Yingping Tian

**Affiliations:** 1 Department of Emergency, The Second Hospital of Hebei Medical University, Shijiazhuang, China; 2 Department of Cardiology, The Second Hospital of Hebei Medical University, Shijiazhuang, China

**Keywords:** lipid metabolism, biomarkers, immune infiltration, pan-cancer, AMI

## Abstract

Background: Acute myocardial infarction (AMI) is associated with high morbidity and mortality, and is associated with abnormal lipid metabolism. We identified lipid metabolism related genes as biomarkers of AMI, and explored their mechanisms of action.

Methods: Microarray datasets were downloaded from the GEO database and lipid metabolism related genes were obtained from Molecular Signatures Database. WGCNA was performed to identify key genes. We evaluated differential expression and performed ROC and ELISA analyses. We also explored the mechanism of AMI mediated by key genes using gene enrichment analysis. Finally, immune infiltration and pan-cancer analyses were performed for the identified key genes.

Results: TRL2, S100A9, and HCK were identified as key genes related to lipid metabolism in AMI. Internal and external validation (including ELISA) showed that these were good biomarkers of AMI. In addition, the results of gene enrichment analysis showed that the key genes were enriched in inflammatory response, immune system process, and tumor-related pathways. Finally, the results of immune infiltration showed that key genes were concentrated in neutrophils and macrophages, and pan-cancer analysis showed that the key genes were highly expressed in most tumors and were associated with poor prognosis.

Conclusions: TLR2, S100A9, and HCK were identified as lipid metabolism related novel diagnostic biomarkers of AMI. In addition, AMI and tumors may be related through the inflammatory immune response.

## INTRODUCTION

Cardiovascular diseases (CVD) cause significant health and economic burdens, with nearly 19 million deaths attributed to CVD by 2020. Acute myocardial infarction (AMI) is one of the most serious CVD. According to a report from National Health and Nutrition Examination Survey (NHANES) 2015 to 2018, the prevalence of AMI was 3.1% in the US, and the mortality was 104,280 in 2019 [1]. In China, AMI-related mortality increased 5.6-fold 11.40 in 1987 to 64.25 in 2014 [2]. Lipid metabolic profiles have been shown to be abnormal in patients with AMI. Several studies have shown that obesity and hyperlipidemia were associated with a shorter life expectancy and could increase cardiovascular mortality and morbidity [3]. In addition, high serum cholesterol and free fatty acids (FFA) are risk factors of cardiovascular disease and independent risk factors for cardiovascular death [4]. Excessive low density lipoprotein cholesterol (LDL-C) builds up in arteries as plaques, resulting in increased risk of AMI. All patients who have experienced AMI should use statins to reduce LDL-C. These findings indicate that lipid metabolism plays a key role in the occurrence and prognosis of AMI.

Regulation of lipid (fatty acid and cholesterol) metabolism is essential for maintaining cell homeostasis, and abnormal lipid metabolism is a key feature of cancer [5]. Recent studies showed that cancer cells regulated lipid metabolism through intracellular carcinogenic signals and the tumor microenvironment. Moreover, abnormal lipid metabolism altered carcinogenic signaling pathways in cancer cells and promoted proliferation, survival, invasion, and metastasis of cancer cells [5]. Other studies have shown that the fatty acid transporters CD36, SLC27, and FABPs were up-regulated in cancers [6]. A study showed that CD36 was associated with poor prognosis of breast cancer, ovarian cancer, gastric cancer, and prostate cancer [7]. Low density lipoprotein receptors (LDLRs) were positively correlated with poor prognosis of small cell lung cancer, breast cancer, and pancreatic cancer [8].

In this study we found that abnormal lipid metabolism was a feature of coronary heart disease (especially AMI) and tumors. We evaluated the role of lipid metabolism related genes in onset and progression of AMI using bioinformatics analysis. We identified key genes as novel diagnostic biomarkers of AMI. Finally, we performed pan-cancer analysis to explore the expression and role of lipid metabolism related genes in tumors to identify possible relationships between AMI and tumors.

## MATERIALS AND METHODS

### Data collection

Acute myocardial infarction-related microarray data were downloaded from GEO database (http://www.ncbi.nlm.nih.gov/geo/). Lipid metabolism related genes were obtained from Molecular Signature Database (https://www.gsea-msigdb.org/gsea/msigdb/index.jsp).

### Weighted gene co-expression network analysis (WGCNA)

Weighted gene co-expression network analysis is a systems biology method for describing the correlation patterns among genes across microarray samples [9]. We analyzed GSE66360 using the WGCNA R package to find gene modules related to AMI. Then, genes closely related to AMI were selected as potential target genes for subsequent analysis. Genes with absolute gene module membership > 0.8 and gene trait significance > 0.2 were intersected with genes related to lipid metabolism, and were identified as hub genes.

### Identification and validation of key genes in AMI

Using WGCNA analysis we identified hub genes that played important roles in development of AMI. The network of co-expression of these genes was constructed using STRING (https://cn.string-db.org/). We then identified key genes using cytoHubba, a plug-in of Cytoscape software. We verified the expression and diagnostic efficacy of key genes in AMI using differential expression and receiver operating characteristic (ROC) curve analysis.

### Patient samples and ethics statement

Ten patients with AMI and 5 healthy controls who visited the department of emergency in the Second Hospital of Hebei Medical University from January to June 2022 were enrolled as the external validation cohort after obtaining approval from the Institute Ethics Committee (Research Ethics Committee of the second hospital of Hebei Medical University, 2021-R495). Informed consent was obtained from all participants.

### Enzyme-linked immunosorbent assay (ELISA)

Double antibody sandwich ELISA was performed. Human anti-TLR2 (E-AB-13835, Elabscience), anti-S100A9 (E-AB-40316, Elabscience), and anti-HCK (E-AB-14119, Elabscience) were coated on the enzyme plate. For detailed experimental steps, refer to previously published articles and product instructions. When the chromogenic substrate (TMB) was added, horseradish peroxidase causes the TMB solution to turn blue, and the solution turns yellow after addition of the termination solution. A microplate reader was used to measure the OD value at 450 nm. The concentrations of TLR2, S100A9, and HCK were proportional to the OD450 values.

### Gene enrichment analysis

After obtaining hub genes and key genes, we explored their possible mechanisms of action using gene enrichment analysis. We performed GO, KEGG, Hallmark analysis, and Metascape analysis (http://metascape.org/gp/index.html#/main/step1). Gene set enrichment analysis (GSEA) was performed using GSEA software (http://software.broadinstitute.org/gsea/).

### Immune infiltration analysis

Single-sample gene set enrichment analysis (ssGSEA) and quanTIseq immune infiltration were performed to estimate the proportion of immunocytes in AMI and the relationship of these immunocytes with key genes. The degree of immune cell infiltration was quantified using enrichment scores calculated using the GSVA (gene set variation analysis) package in R. Spearman correlation analysis was used to evaluate the relationship between expression levels of key genes and immunocyte and immunological processes.

### Pan-cancer analysis

We downloaded sequencing data from 34 tumor types from TCGA (https://www.cancer.gov/about-nci/organization/ccg/research/structural-genomics/tcga). Sequencing information from the corresponding normal tissues was obtained from the GETx database (https://gtexportal.org/). The R language was used for data standardization prior to data merging. Expression levels of key genes were extracted from the data. Wilcoxon analysis was performed to compare differences in expression between tumors and corresponding normal tissues. We also collected data regarding survival time and survival status of patients with tumors. Cox regression analysis was used to evaluate the influence of key genes on prognosis.

### Statistical analysis

Results are shown as the mean ± standard deviation. Levels of key genes levels in AMI vs. control tissues were compared using the Wilcoxon signed-rank test. Spearman’s rank test was used to assess correlation between expression levels of key genes and immunocyte and immunological processes. The ROC curve was used to estimate the diagnostic value of key genes. Statistical analyses were performed using SPSS 24.0 (IBM), GraphPad Prism 8, and R 4.0.1. *P* < 0.05 was considered statistically significant.

### Data availability statement

Publicly available datasets were analyzed in this study. All the raw data used in this study are available in the public GEO database (https://www.ncbi.nlm.nih.gov/geo/; Accession numbers: GSE66360, GSE48060, and GSE60993).

## RESULTS

### Workflow diagram and RNA microarray profiles collection

**Figure 1:**
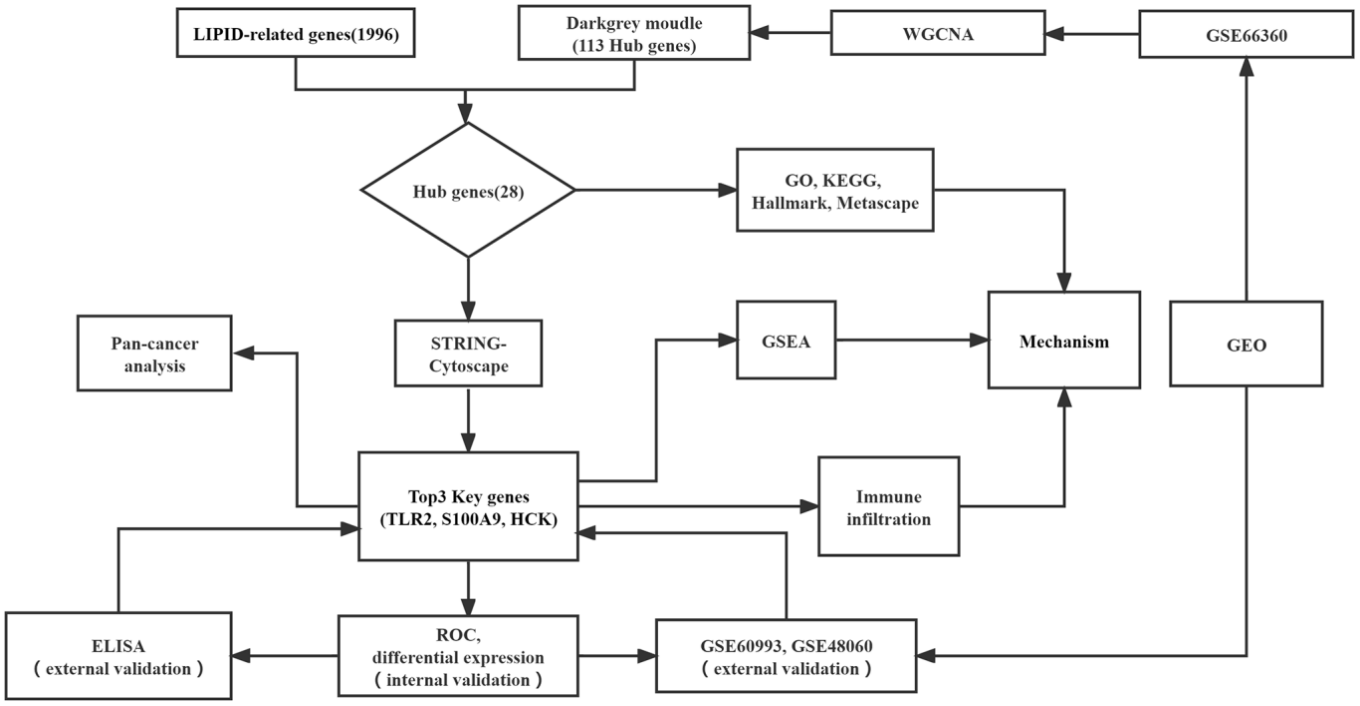
Flowchart of this study.

The workflow diagram for our study is shown in Figure 1. Three RNA expression datasets (GSE66360, GSE48060, and GSE60993) were downloaded from GEO, which platform were GPL570, GPL570, and GPL6884 sequentially. GSE66360, which included 49 patients with AMI and 50 healthy controls, selected as the test cohort. GSE48060 included 31 patients with AMI and 21 healthy controls and GSE60993 included 17 patients with AMI and 7 controls, and were used as validation sets (Table 1). 10 patients with AMI and 5 controls visiting the department of emergency in the Second Hospital of Hebei Medical University from January to June 2022 were enrolled as the external validation cohort.

**Table 1:** Base situation of data sets.

**Data set**	**AMI**	**Control**	**Platform**
GSE66350	49	50	GPL570
GSE48060	31	21	GPL570
GSE60993	17	7	GPL6884

### Identification of hub genes related to lipid metabolism in AMI

**Figure 2:**
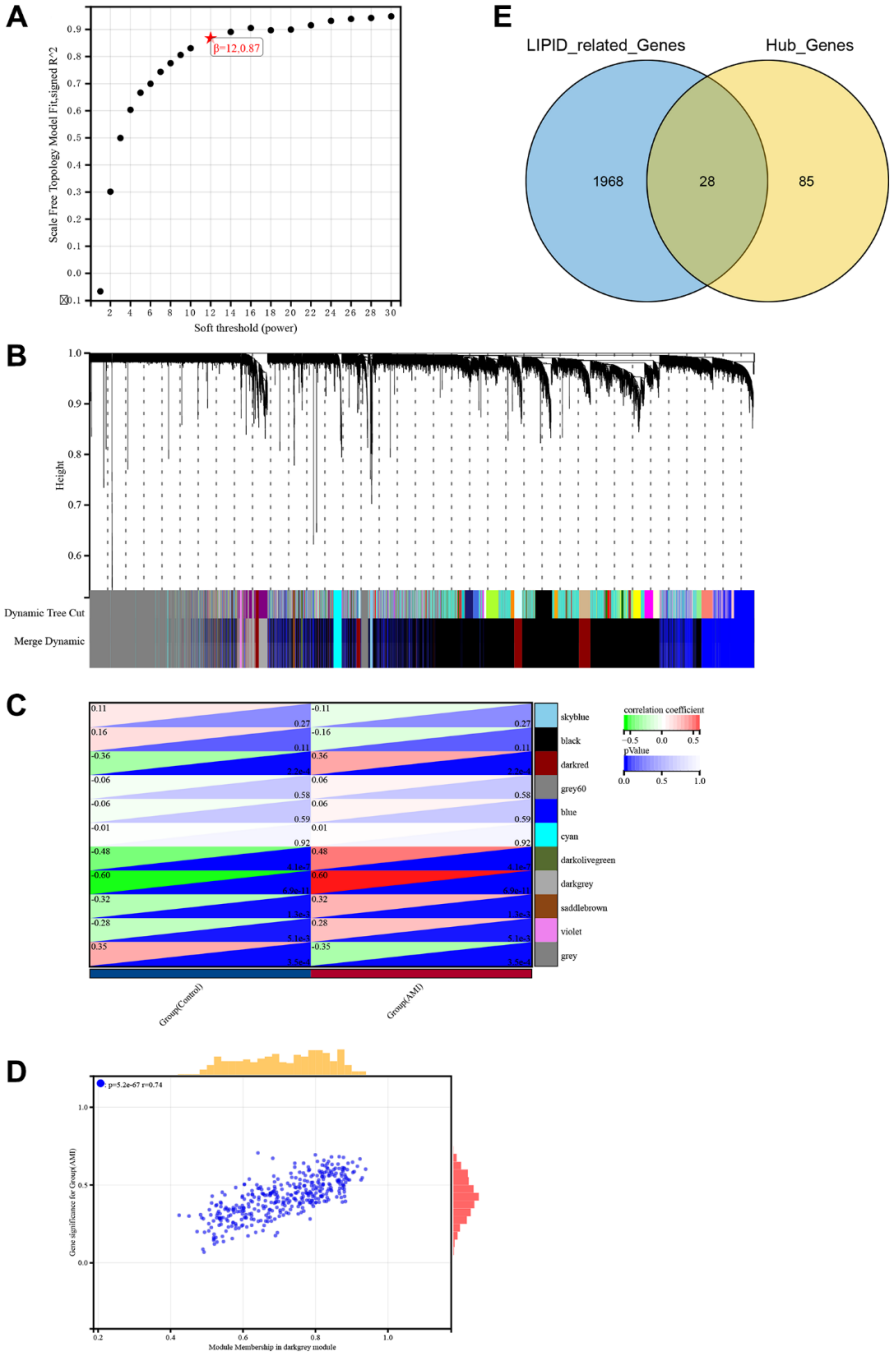
**Identification of hub genes related to lipid metabolism in AMI using WGCNA.** (**A**) Soft threshold screening. (**B**) Eleven modules shown by tree diagram. (**C**) Correlation heat map showed the relationship between 11 modules and groups (AMI and control). (**D**) Scatter diagram showed the correlation between the dark grey module and AMI. (**E**) Venn diagram showed the intersection of 113 genes and lipid metabolism related genes.

Weighted correlation network analysis (WGCNA) is a systematic biological method performed to screen genes associated with clinical features between different cases. It is an effective means of identification of biomarkers. We used GSE66360 to conduct WGCNA. Pearson’s correlation matrices and average linkage were computed for each pair-wise gene. We then constructed a weighted adjacency matrix using a power function. The soft-thresholding parameter β was 12.087 (Figure 2A). To identify gene modules according to their expression profiles, average linkage hierarchical clustering was performed using the topological overlap matrix (TOM) based dissimilarity measure on a module with a minimum of 30 genes. We set the sensitivity of the module to 3 and merged the modules with distances less than 0.25. Eleven co-expression modules were obtained (Figure 2B). The relationships between modules and clinical phenotypes (AMI and control groups) resulted in identification of the dark grey module, with a coefficient of correlation of 0.6 and *P*-value of 6.9e-11 (Figure 2C, 2D). In accordance with the cut-off criteria (MM > 0.8, GS > 0.1, weight coefficient >0.1), 113 WGCNA-related genes with good connectivity were identified in the dark grey module. We then screened 1,996 genes related to lipid metabolism using the Molecular Signatures Database. Finally, we found that 28 hub genes identified in WGCNA were lipid metabolism-related genes (Figure 2E).

### Functional enrichment analysis of hub genes set

To illustrate hub genes, we used GO, KEGG, and hallmark enrichment analyses. Supplementary Figure 1A–1C shows the top ten enriched GO terms. Hub genes were mainly enriched in lipid metabolism (response to lipid, response to lipopolysaccharide, cellular response to lipopolysaccharide) and inflammatory response (response to molecule of bacterial origin, response to bacterium, cellular response to molecule of bacterial origin) in BP. Hub genes were enriched in secretory granule, secretory vesicle, cytoplasmic vesicle membrane, vesicle membrane, whole membrane, plasma membrane part, integral component of plasma membrane, and tertiary granule membrane in CC. Hub genes were enriched in lipid metabolism (lipopolysaccharide binding, lipopolysaccharide receptor activity, lipopeptide binding) and immune responses (signaling pattern recognition receptor activity, pattern recognition receptor activity, Toll-like receptor binding) in MF. Hub genes were primarily enriched in fat digestion and absorption, B cell receptor signaling pathway, IL-17 signaling pathway, rheumatoid arthritis, cholesterol metabolism, and phagosome pass in KEGG analysis (Supplementary Figure 1D). Moreover, hub genes were enriched in inflammatory response (TNFα signaling via NF-κB, Complement, IL6 jak stat3 signaling) and lipid metabolism (bile acid metabolism, fatty acid metabolism) in hallmark analysis (Supplementary Figure 1E).

Metascape integrates data from more than 40 biological information databases to allow for identification of potential mechanisms of hub genes. The results of Metascape analysis showed that hub genes were mainly enriched in lipid metabolism (response to lipopolysaccharide, response to fatty acid, lipid localization), inflammatory response related pathways (regulation of interleukin-6 production, cellular response to cytokine stimulus, regulation of cell-cell adhesion), and immune-related pathways (regulation of phagocytosis, phagocytosis, response to interferon-gamma) (Supplementary Figure 2A, 2B).

### Construction of a PPI network and selection key genes

**Figure 3:**
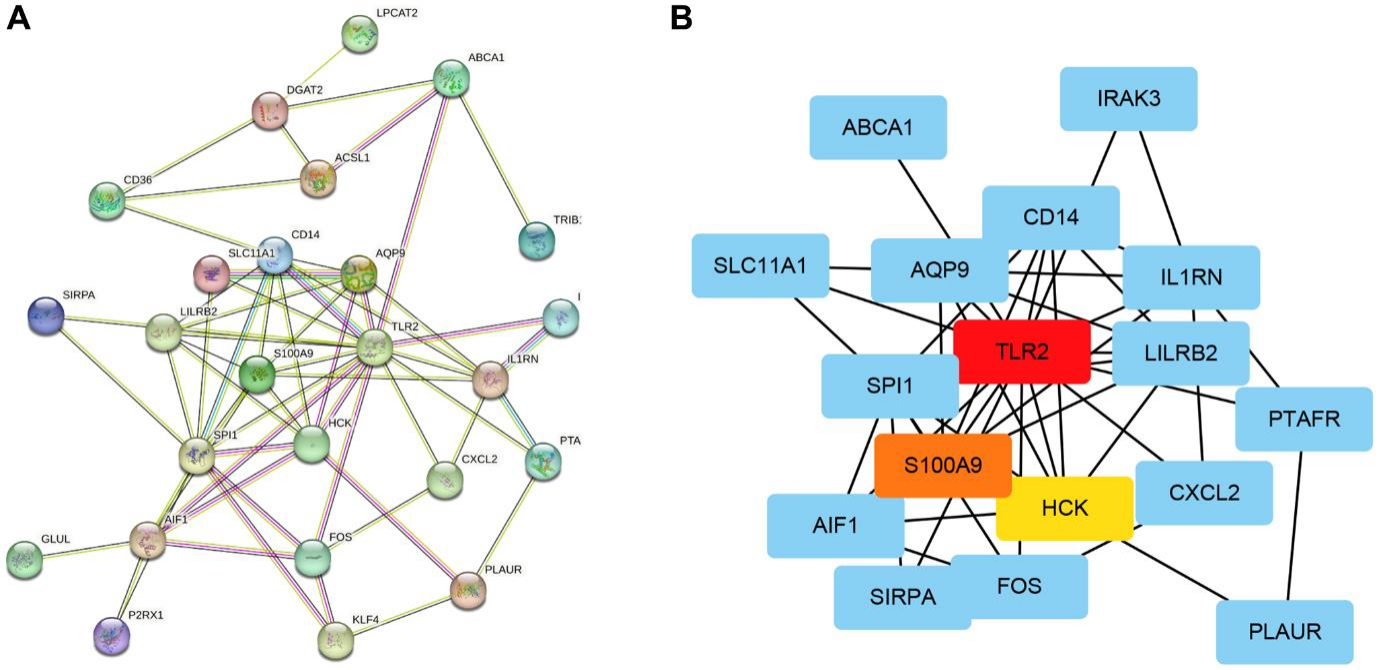
**Construction of a PPI network and selection key genes.** (**A**) Network construction of 28 hub genes using STRING. (**B**) Identification of 3 key genes using Cytoscape software.

We established 28 hub genes and used the STRING database Version 11.5 (https://cn.string-db.org/) to construct a PPI network (Figure 3A). The network of 28 genes was analyzed using Cytoscape to calculate the top 3 genes ranked by MCC. As seen in Figure 3B, TLR 2, S100A9, and HCK were the top 3 key genes selected for further analysis.

### Internal and external validation of key genes

**Figure 4:**
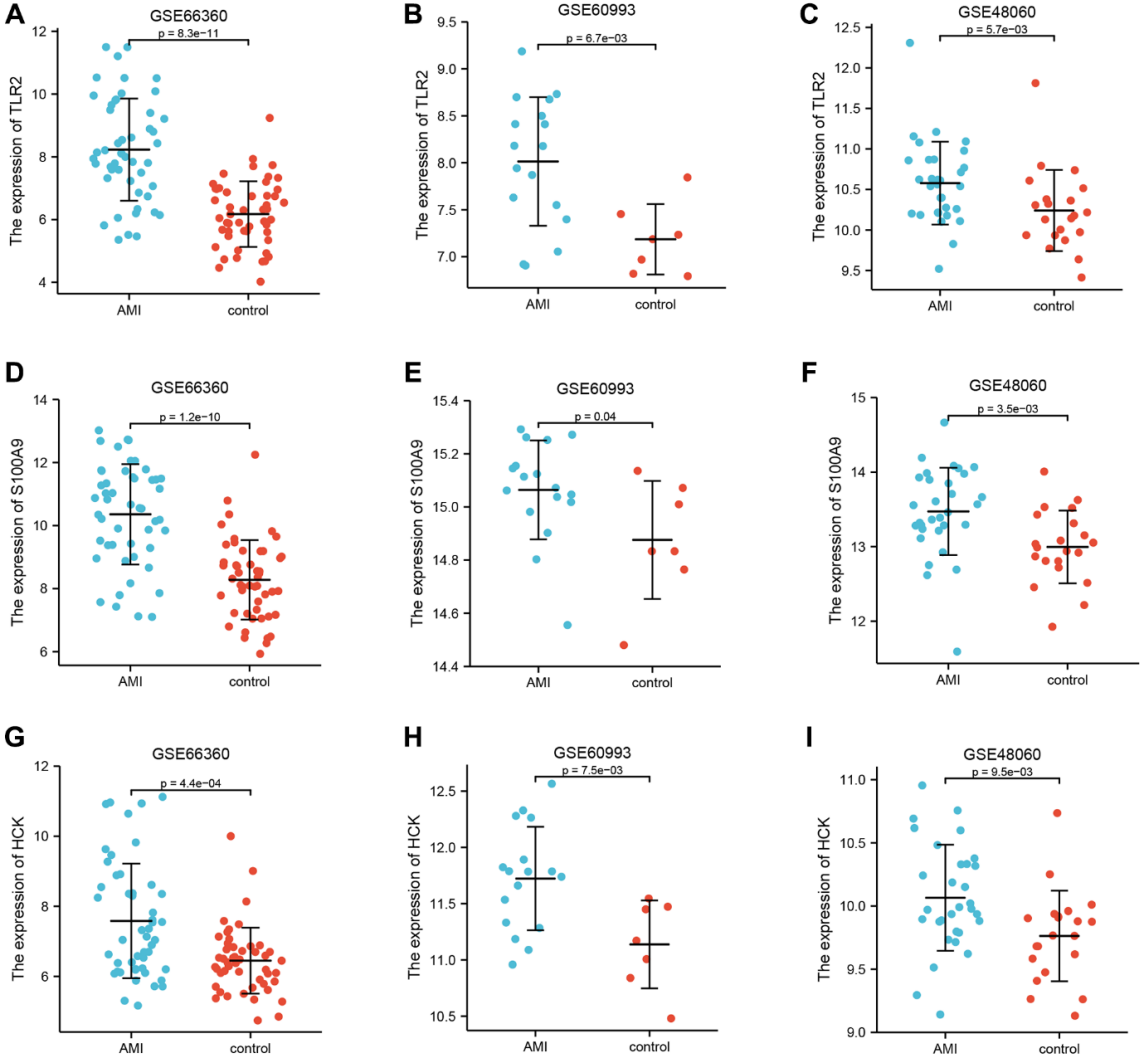
**Differential expression of key genes.** (**A**–**C**) Differential expression of TLR2 between AMI and control in GSE66360, GSE60993, and GSE48060. (**D**–**F**) Differential expression of S100A9 between AMI and control in GSE66360, GSE60993, and GSE48060. (**G**–**I**) Differential expression of HCK between AMI and control in GSE66360, GSE60993, and GSE48060.

**Figure 5:**
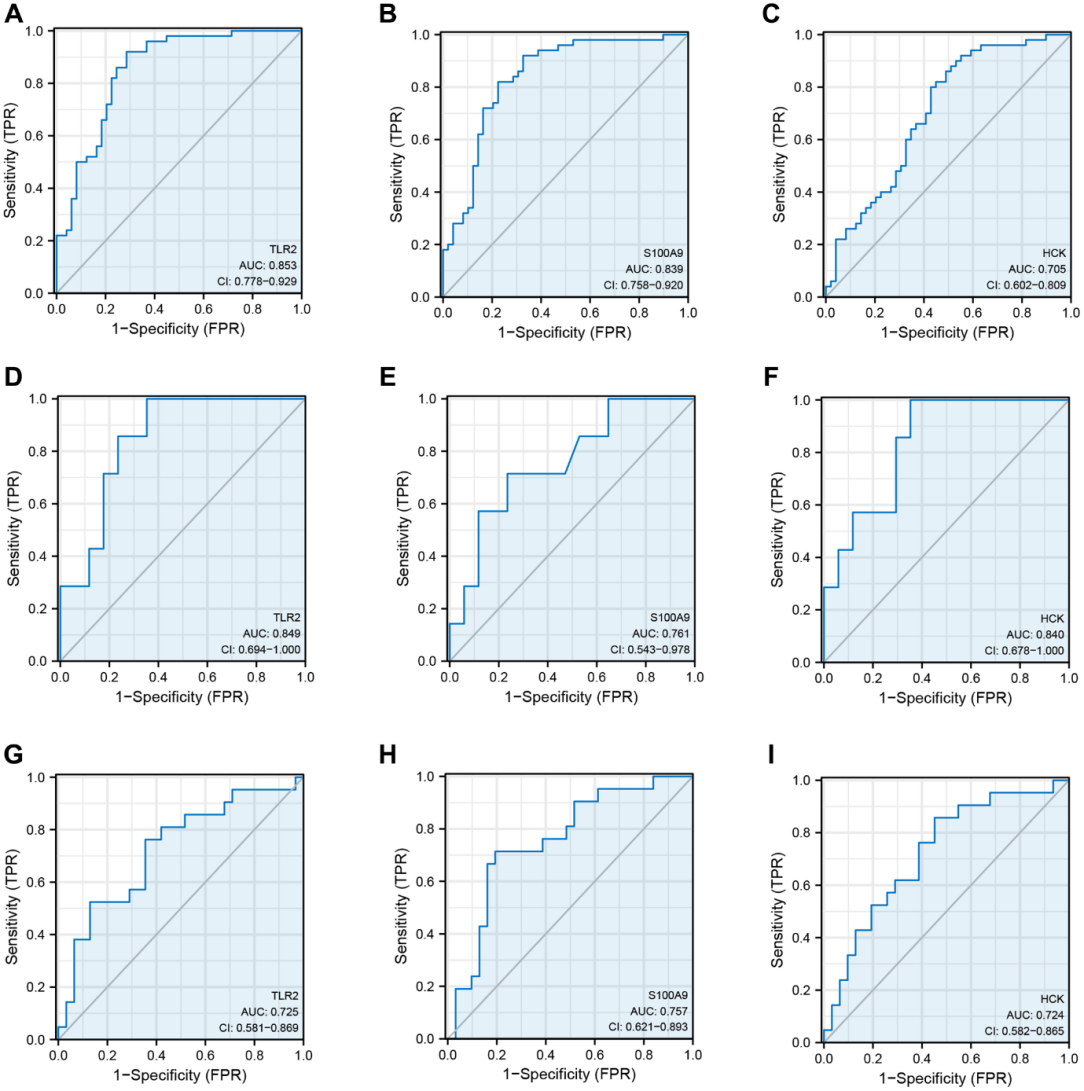
**Receiver operating curve analysis of key genes.** (**A**–**C**) ROC curve showing the diagnostic value of TLR2, S100A9, and HCK in GSE66360. (**D**–**F**) ROC curve showing the diagnostic value of TLR2, S100A9, and HCK in GSE60993. (**G**–**I**) ROC curve showing the diagnostic value of TLR2, S100A9, and HCK in GSE48060.

GSE66360 was used as the internal cohort to evaluate the expression and diagnostic value of key genes. As shown in Figure 4A–4I, the expression levels of key genes were higher in AMI than those in the control group. To estimate the diagnostic value of TLR2, S100A9, and HCK, the areas under ROC curves (AUC) were calculated. Figure 5A–5C and Table 2 show the AUC for TLR2 was 0.853 (95% CI, 0.778–0.929, *p* = 0.000), 0.839 for S100A9 (95% CI, 0.758–0.920, *p* = 0.000), 0.705 for HCK (95% CI, 0602–0,809, *p* = 0.000). These results showed that these key genes had good diagnostic value. These results were confirmed in the external validation cohorts (GSE60993 and GSE48060) (Figure 5D–5I and Table 2).

**Table 2:** ROC of key genes in data sets.

**Data set**	**Key genes**	**AUC**	**Cut-off value**	**Sensitivity**	**Specificity**	**95% CI**
	TLR2	0.853	7.478	0.714	0.920	0.778–0.929
GSE66360	S100A9	0.839	9.246	0.776	0.820	0.758–0.920
	HCK	0.705	6.893	0.571	0.800	0.602–0.809
	TLR2	0.725	10.387	0.762	0.645	0.581–0.869
GSE48060	S100A9	0.757	13.183	0.714	0.806	9.621–0.893
	HCK	0.724	9.965	0.857	0.548	0.582–0.865
GSE60993	TLR2	0.849	7.857	0.647	1.000	0.694–1.000
	S100A9	0.761	15.014	0.765	0.867	0.543–0.978
	HCK	0.840	11.604	0.648	1.000	0.678–1.000

### Key genes were assessed by ELISA

**Figure 6:**
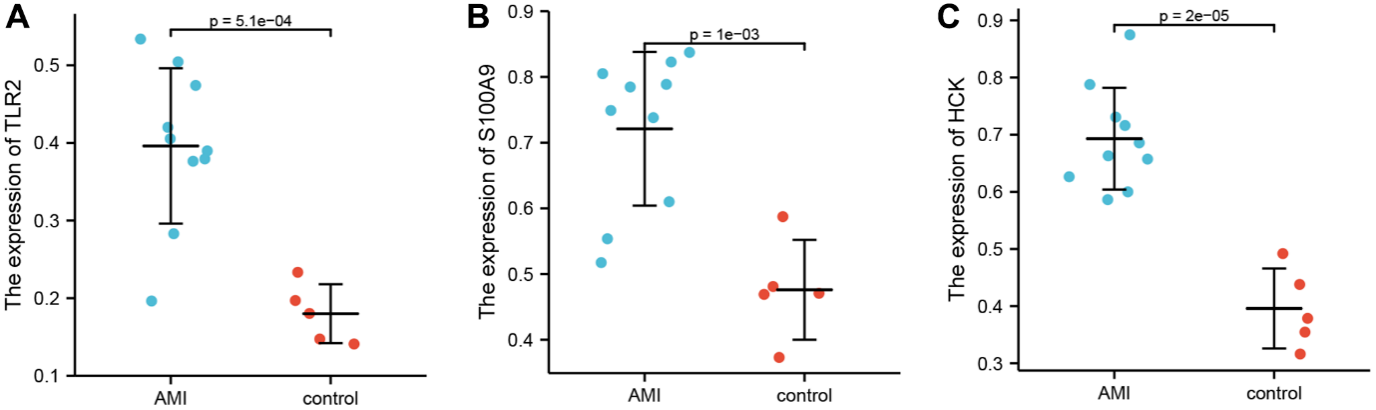
**Differential expression of TLR2, S100A9, and HCK using ELISA.** (**A**) Differential expression of TLR2 between AMI and control. (**B**) Differential expression of S100A9 between AMI and control. (**C**) Differential expression of HCK between AMI and control.

Enzyme-linked immunosorbent assay was used to quantitate the expression levels of TLR2, S100A9, and HCK in serum. Ten patients with AMI and five controls were enrolled. The results showed that the levels of TLR2, S100A9, and HCK were significantly higher in the AMI group than those in the control group (Figure 6).

### Functional enrichment analyses of key genes set

**Figure 7:**
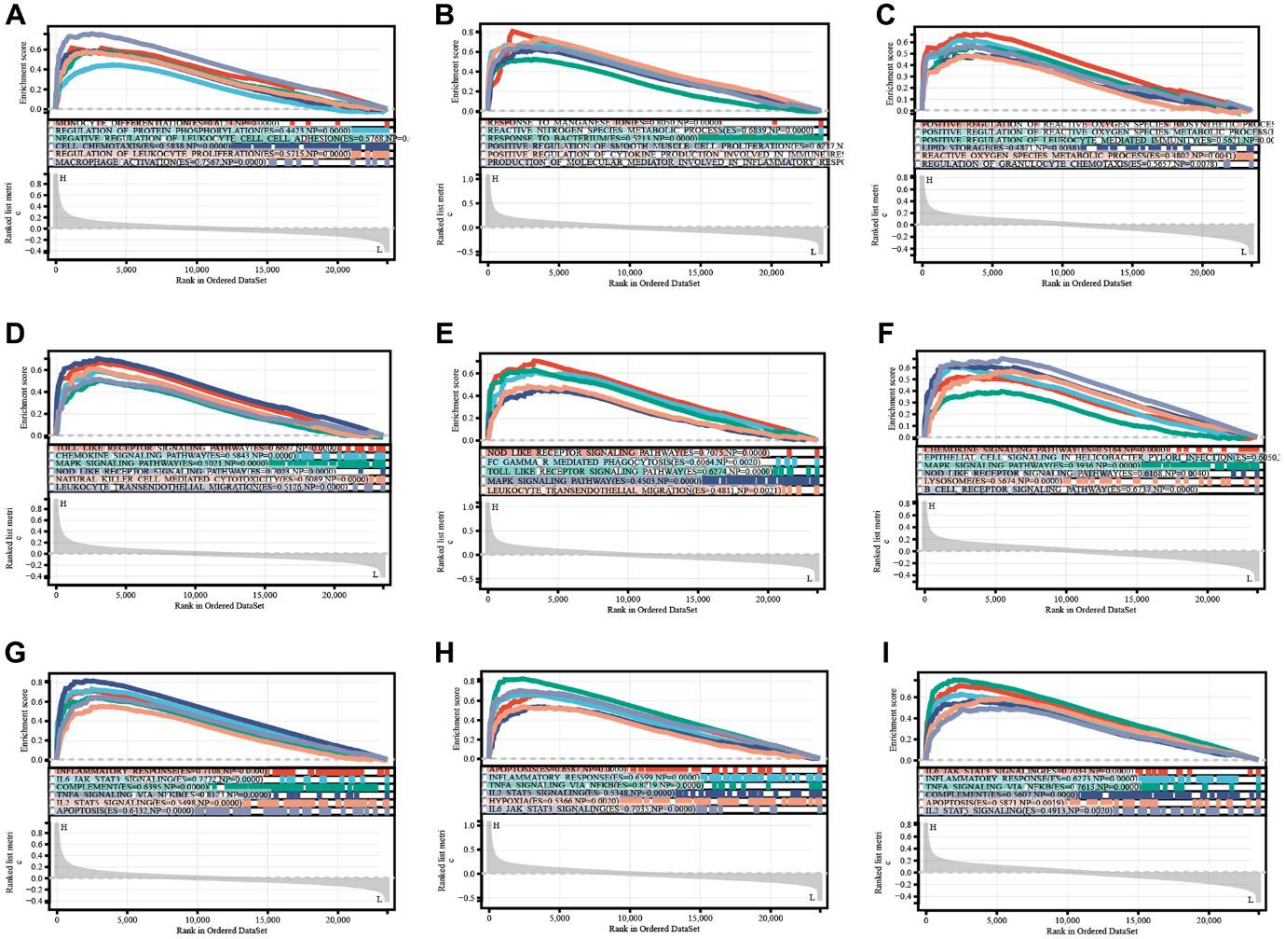
TLR2, S100A9, and HCK enrichment analyses of BP (**A**–**C**), KEGG (**D**–**F**), and Hallmark (**G**–**I**).

Gene set enrichment analysis was performed to determine the mechanisms of key genes. The significantly enriched terms are shown in Figure 4. Key genes were predominantly enriched in modulation of the immune and inflammatory responses (REGULATION_OF_INFLAMMATORY_RESPONSE, POSITIVE_REGULATION_OF_LEUKOCYTE_MIGRATION, REGULATION_OF_IMMUNE_SYSTEM_PROCESS, MONOCYTE_DIFFERENTIATION, CELL_CHEMOTAXIS, REGULATION_OF_LEUKOCYTE_PROLIFERATION, and MACROPHAGE_ACTIVATION, REGULATION_OF_CELL_CELL_ADHESION) by GO-BP (Figure 7A–7C, Supplementary Table 1). They were mostly enriched in inflammation, immune, and tumor related pathways (TOLL_LIKE_RECEPTOR_SIGNALING_PATHWAY, MAPK_SIGNALING_PATHWAY, NOD_LIKE_RECEPTOR_SIGNALING_PATHWAY, FC_EPSILON_RI_SIGNALING_PATHWAY, FC_GAMMA_R_MEDIATED_PHAGOCYTOSIS, and PATHWAYS_IN_CANCER) via KEGG analysis (Figure 7D–7F, Supplementary Table 1). Moreover, these genes were enriched in inflammatory response, apoptosis, and tumor (IL6_JAK_STAT3_SIGNALING, TNFA_SIGNALING_VIA_NFKB, COMPLEMENT, APOPTOSIS, IL2_STAT5_SIGNALING, KRAS_SIGNALING_UP) using hallmark analysis (Figure 7G–7I, Supplementary Table 1).

### Immune infiltration in AMI and key genes

**Figure 8:**
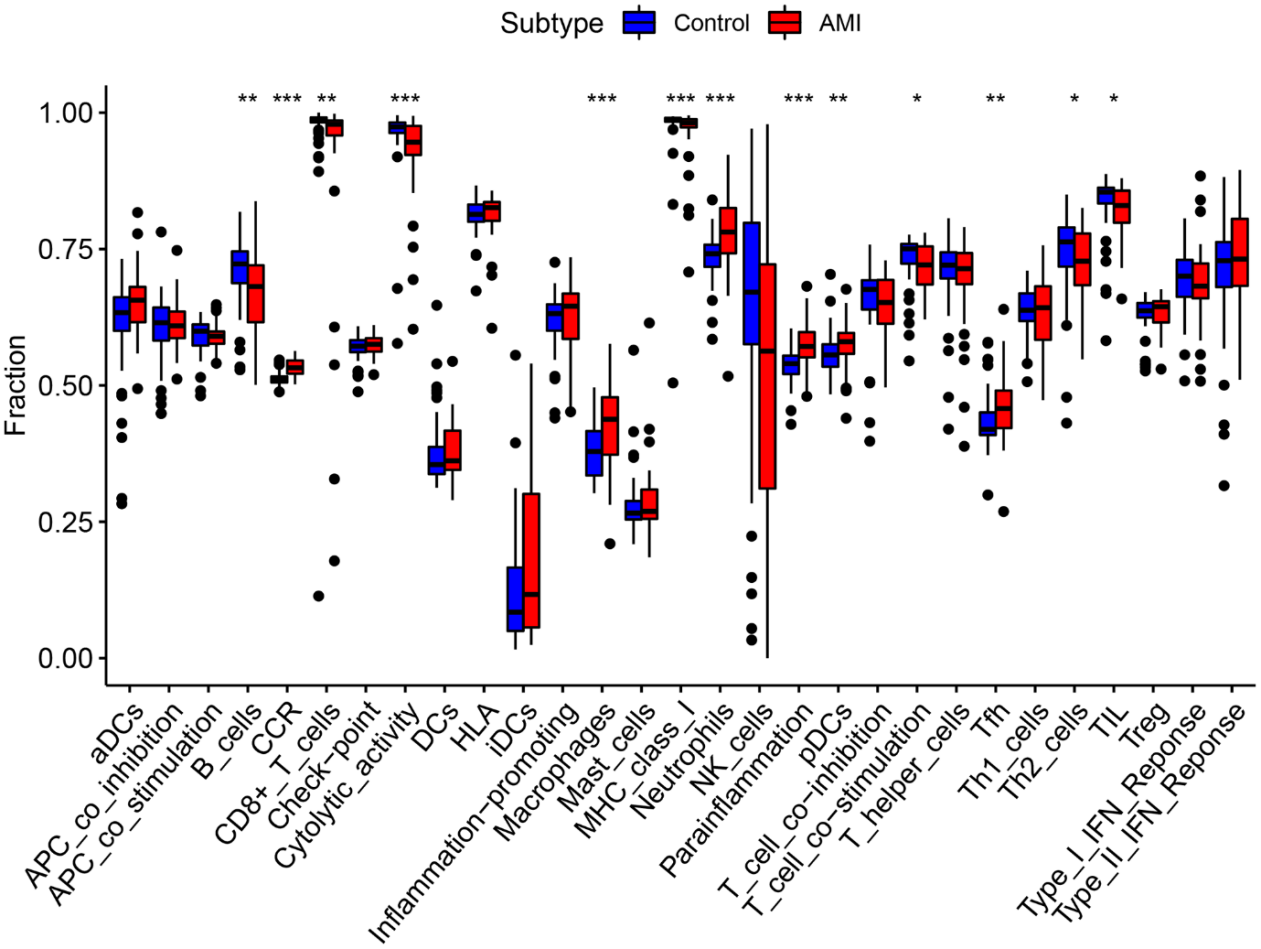
Comparison of immune infiltration cells and processes between AMI and control.

**Figure 9:**
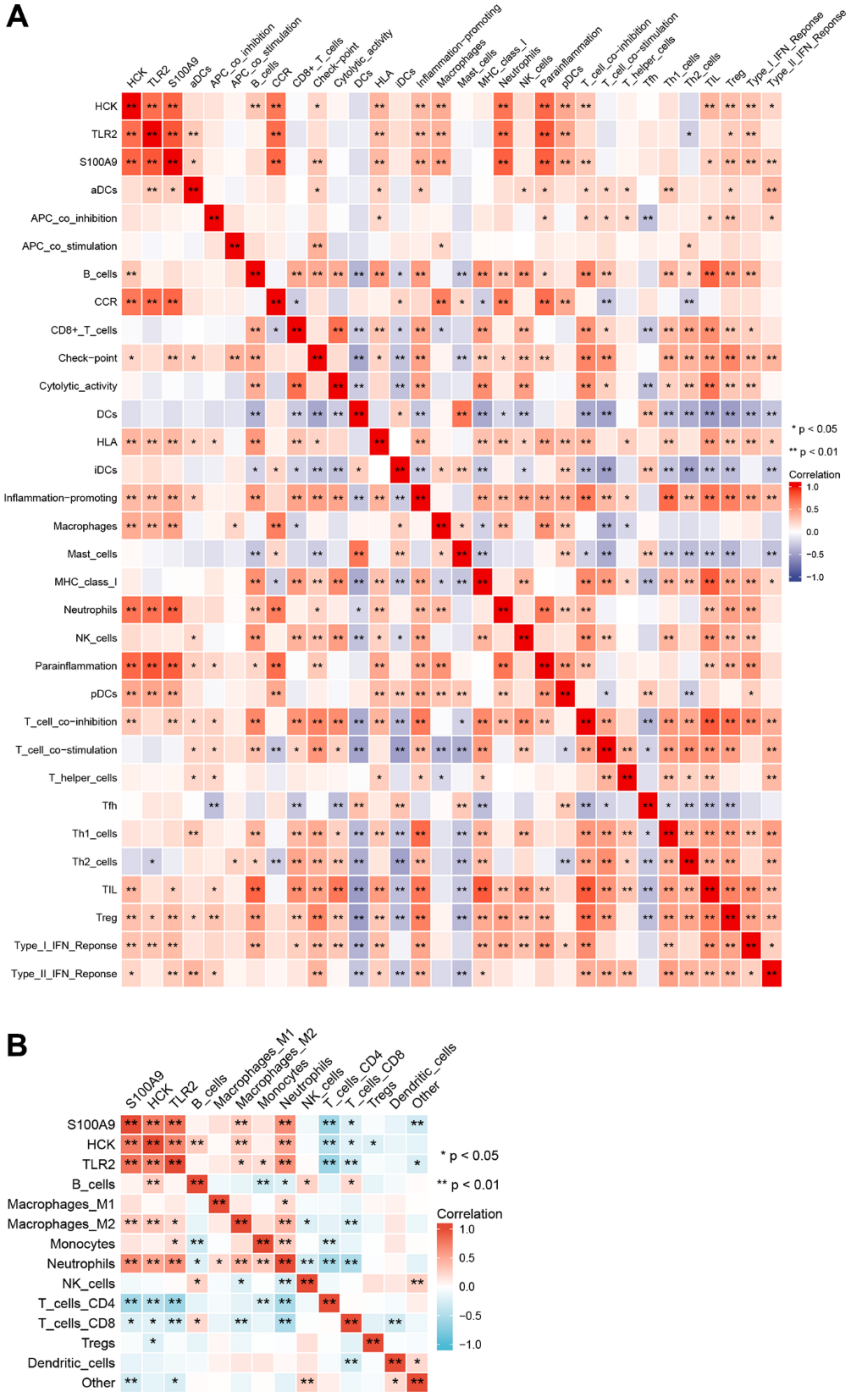
**Correlation among immune infiltration cells, processes, and key genes.** (**A**) ssGSEA immune infiltration analyses. (**B**) quanTIseq immune infiltration analyses.

We performed ssGSEA and quanTIseq immune infiltration analyses on the RNA expression profiles. In ssGSEA, infiltrating cells and processes such as CCR, Macrophages, Neutrophils, Parainflammation, pDCs, and Tfh were higher in the AMI group than in the control group. However, B_cells, Cytolytic_activity, and T_cell_co-inhibition were lower in the AMI group than in the control group (Figure 8). In addition, we showed that TLR2 was positively associated with aDCs and Th2_cells, S100A9 was positively associated with aDCs, Check-point, T_cell_co_inhibition, and HCK was positively associated with B_cells and T_cell_co_inhibition. Key genes were all positively correlated with the infiltration of Macrophages, Neutrophils, Treg, pDCs, HLA, CCR, inflammation-promoting, and Parainflammation (Figure 9A). In quanTIseq, we identified that the key genes were positively correlated with the infiltration of Macrophages_M2, Neutrophils, and negatively correlated with T_cell_CD4, T_cell_CD8 (Figure 9B).

### Pan-cancer analysis of key genes

**Figure 10:**
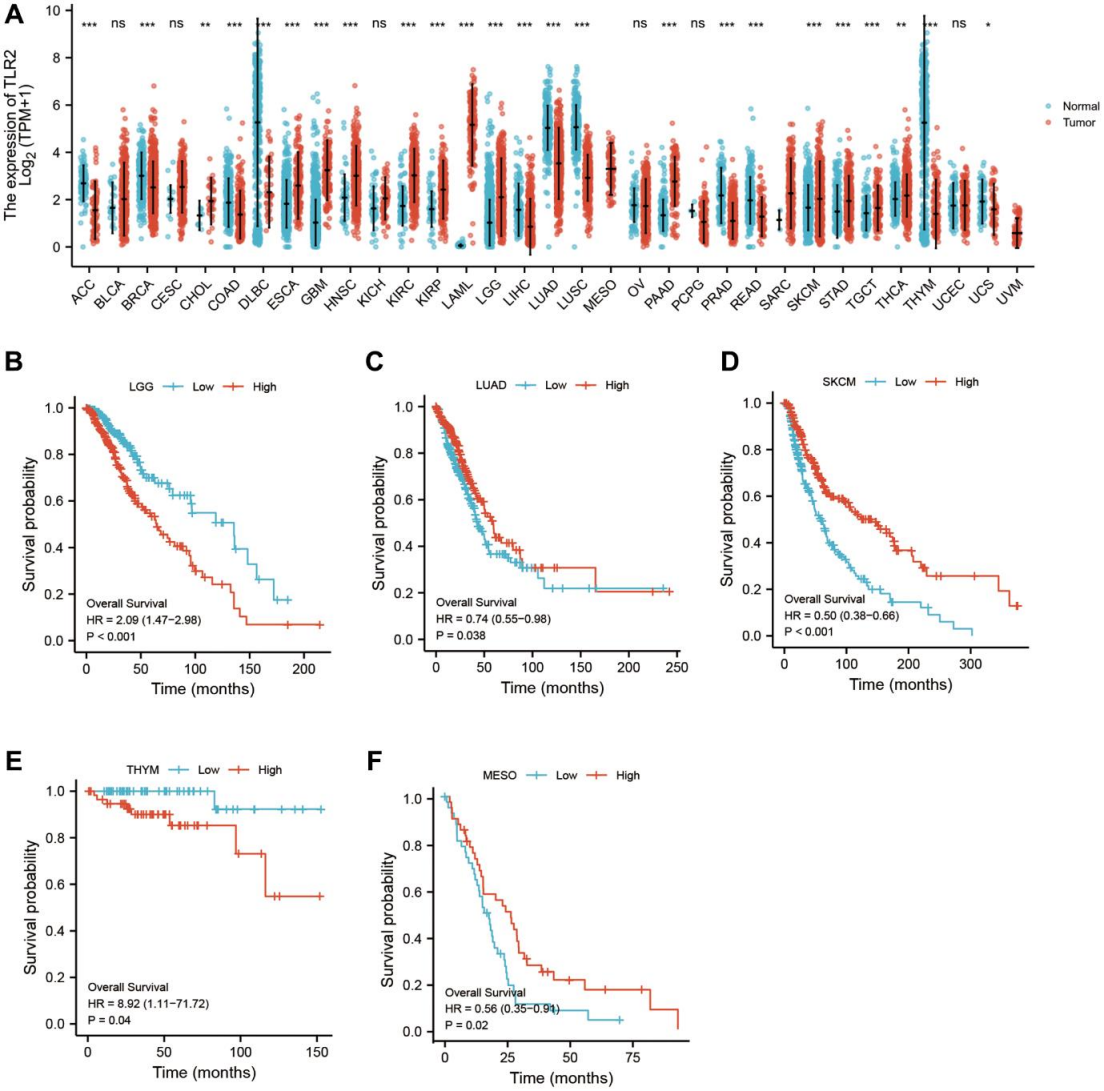
Pan-cancer analysis showed that TLR2 was highly expressed in most tumors (**A**) and its effect on prognosis (**B**–**F**).

**Figure 11:**
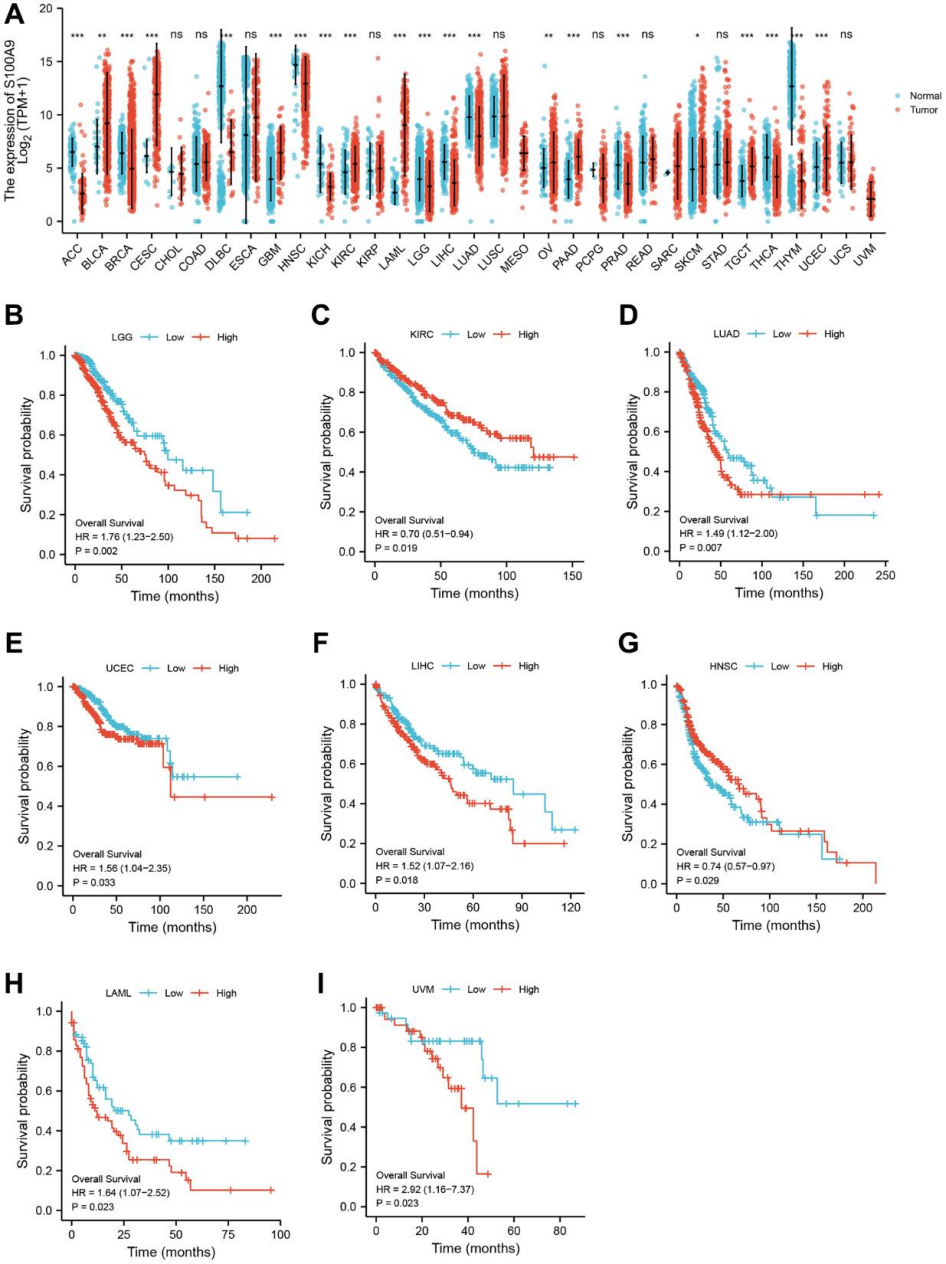
Pan-cancer analysis showed that S100A9 was highly expressed in most tumors (**A**) and its effect on prognosis (**B**–**I**).

**Figure 12:**
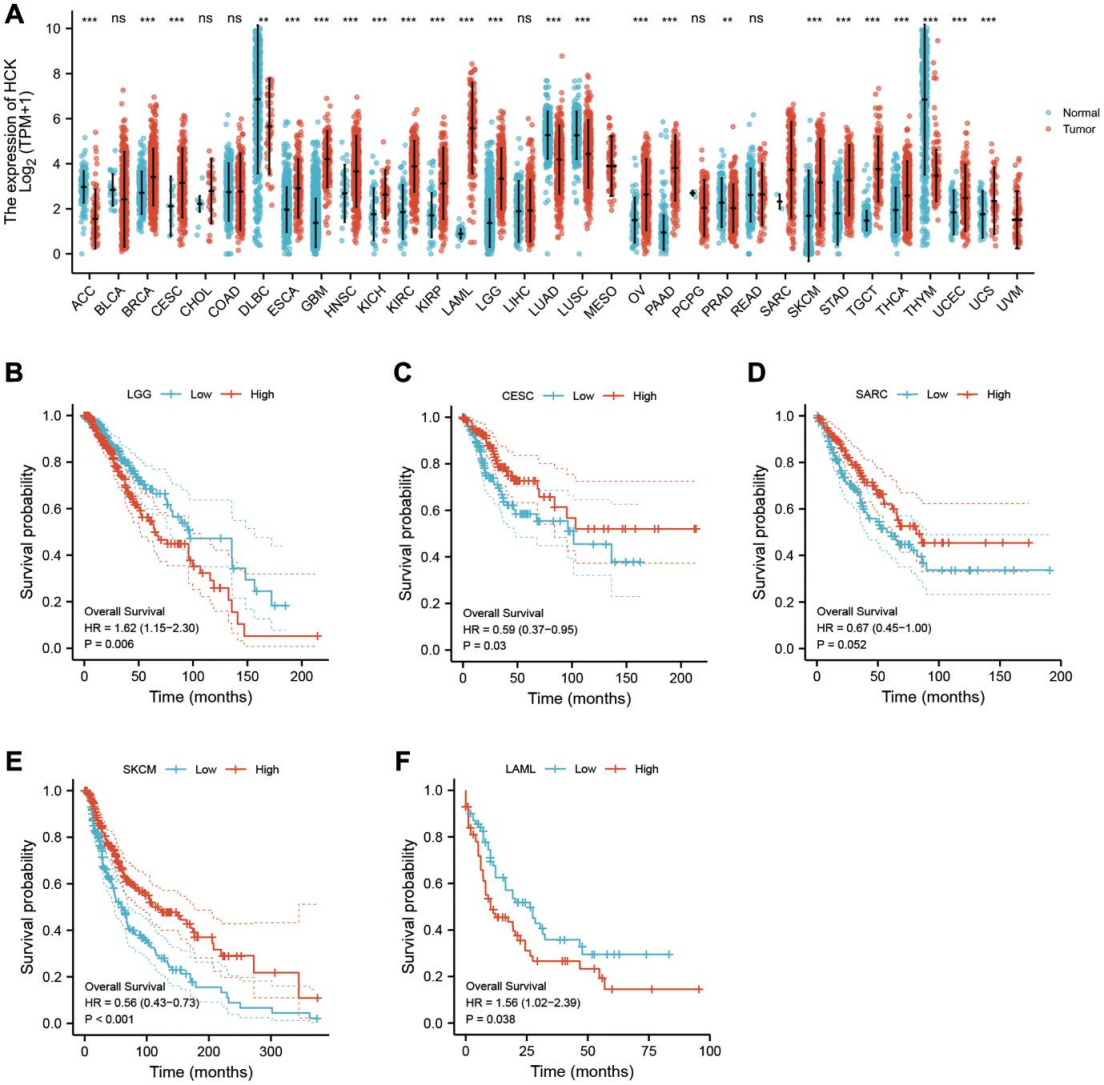
Pan-cancer analysis showed that HCK was highly expressed in most tumors (**A**) and its effect on prognosis (**B**–**F**).

Using pan-cancer analysis, we found that TLR2 was highly expressed in most tumors and survival analysis also showed this key gene was closely related to poor prognosis of some tumor types, such as LGG (Lower Grade Glioma), THYM (Thymoma). While, TLR2 was negatively related to poor prognosis of LUAD (Lung adenocarcinoma), SKCM (Skin Cutaneous Melanoma), and MESO (Mesothelioma) (Figure 10A–10F). S100A9 was highly expressed in most tumors and survival analysis also showed this key gene was closely related to poor prognosis of some tumor types, such as LGG, KIRC (Kidney renal clear cell carcinoma), LUAD, UCEC (Uterine Corpus Endometrial Carcinoma), LIHC (Liver hepatocellular carcinoma), LAML (Acute Myeloid Leukemia) and UVM (Uveal Melanoma). While, S100A9 was negatively related to poor prognosis of HNSC Head and Neck squamous cell carcinoma), SKCM, and MESO (Figure 11A–11I). HCK was highly expressed in most tumors and survival analysis also showed this key gene was closely related to poor prognosis of some tumor types, such as LGG, and LAML. While, HCK was negatively related to poor prognosis of CESC, SARC (Sarcoma), and SKCM (Figure 12A–12F). These results suggested that the key genes identified in our study may be involved in development of AMI and in progression of tumors.

## DISCUSSION

Cardiovascular diseases and tumors are diseases that pose major threats to human health worldwide. Lipid metabolism plays a key role in the onset and progression of both diseases. Moreover, patients with cardiovascular diseases have a 76% increased risk of cancer [10]. Some studies have shown that cancer is a major cause of death in patients with cardiovascular diseases due to non-cardiovascular causes [11]. Acute myocardial infarction is a serious disease with high morbidity and mortality that causes a great economic burden to society. Researchers have shown that the 30-day mortality rate of patients with AMI patients is 7.8% due to various acute and subacute complications [12].

In this study, we downloaded three RNA expression profiles from patients with AMI from the GEO online database, including GSE66360, GSE40860, andGSE60993. TLR2, S100A9, and HCK were identified as key genes related to lipid metabolism by WGCNA. Moreover, internal and external verification were performed to evaluate the diagnostic and predictive value of key genes. Then, we evaluated the mechanisms of development of AMI through enrichment and immune infiltration analyses. Finally, immune infiltration was mainly comprised of neutrophils, and recent studies have shown that neutrophils are critical in the tumor microenvironment and promote tumor development [13]. Therefore, we performed pan-cancer analysis of key genes to explore their roles in AMI and tumors.

The results of ELISA and bioinformatics analyses showed that the expression levels of TLR2, S100A9, and HCK were significantly higher in patients with AMI than those in healthy controls. Toll-like receptors (TLRs) could play a critical role in innate immunity through the NF-κB and MAPK pathways. Toll-like receptors recognize pathogens and induce production of proinflammatory cytokines and upregulate costimulatory molecules within the innate immune system [14]. Many studies have investigated the expression of surface TLR2 in immune cells, including neutrophils, macrophages, B cells, T cells, NK cells, and DC cells. TLR2 signaling could benefit against infection through promotion of immune cell activation. In contrast, dysfunctional TLR2 signaling leads to hyperactive inflammatory responses that could be detrimental in inflammatory and autoimmune diseases [15]. Pro-inflammatory cytokines are elevated in the AMI environment, and TLR2 on the surface of monocytes may be involved in this increase. [16]. Monocytes may participate in AMI pathogenesis through induction of the Thl-type response through TLR2 [17]. In this study, TLR2 was highly expressed in AMI, which indicating that TLR2 could play a vital role in inflammation and the immune response in AMI.

S100A9, a multifunctional calcium-binding protein belonging to the S100 family, plays a significant role in regulating inflammatory and immune responses. Yize Sun [18] identified S100A9 as a promoter of macrophage inflammation, and blockade of S100A9 ameliorated reduced cardiac function [19]. Recent studies have shown that S100A9 was significantly upregulated in the myocardium immediately after ischemia, which indicated that S100A9 was associated with the initial response to ischemic injury [20]. High levels of S100A9 within 24 h after AMI was associated with a high risk of adverse cardiovascular events [21]. We showed that the expression of S100A9 was up-regulated in patients with AMI using bioinformatics analysis. Therefore, S100A9 is a biomarker for diagnosis of AMI and could be a potential therapeutic target for AMI.

HCK, a member of the non-receptor protein tyrosine kinase (SFK) family, is involved in innate immune response and plays a vital role in phagocytosis and cell function [22]. HCK plays a key role in phagocytosis in macrophages. Defective phagocytosis could promote a persistent proinflammatory state, resulting in impaired heart function. Increased expression of HCK has been shown in pancreatic cancer, colorectal cancer, gastric cancer, and other solid malignant tumors [23]. Other studies have shown that overexpression of HCK was involved in the onset, progression and prognosis of tumors [24]. Our results showed that HCK was significantly upregulated in patients with AMI, which was verified in validation data sets and in *in vitro* experiments. These results indicated that HCK may be associated with tumors and AMI.

Enrichment analysis showed that the identified key genes were enriched in inflammatory and immune response-related regulation pathways, and also in tumor related pathways (ROS, apoptosis, KRAS pathway).

In AMI, apoptotic cardiomyocytes in the infarcted area produce a strong inflammatory cascade. Appropriate inflammatory response is critical to repair of heart tissue, but excessive inflammation results in adverse ventricular remodeling and heart failure. Excessive inflammation is characterized by release a large number of DAMP-related molecules (DAMPs) from apoptotic cardiomyocytes, resulting in release of pro-inflammatory cytokines (TNF-α, IL-1β, and IL-6), and infiltration of abundant neutrophils into the infarcted area. Neutrophils are important cells in innate immunity, and can infiltrate coronary plaques and infarcted myocardium, resulting in tissue damage through release of matrix degrading enzymes and ROS. In an animal model of AMI, the average infarct size was reduced 43% in a group that received anti-neutrophil treatment, which prevented neutrophil infiltration into the infarct area [25]. Furthermore, reduction of inflammatory infiltration to the infarct area could promote infarct healing [26]. Studies have shown that up-regulation of neutrophils was associated with mortality in AMI. Local myocardial inflammation and systemic inflammation have been detected in AMI, and many inflammatory factors, chemokines, and components of the complement system showed abnormal expression [27]. Inflammatory cells promote removal of necrotic cells and tissue repair by regulating myofibroblasts and vascular cells, but may also contribute to abnormal fibrotic remodeling, increased cardiomyocyte apoptosis, and adverse events. Various complex pathways were involved in regulation of inflammation and immune response, including regulation of leukocyte proliferation, chemotaxis, migration, and adhesion, which were enriched with the key genes identified in our study. Systemic inflammatory markers are predictors of severe adverse outcomes in patients with AMI. In addition, the key genes in this study were also enriched in ROS and apoptosis. Reactive oxygen species are an inevitable by-product of mitochondrial oxidative phosphorylation and an important driver of myocardial injury. Reactive oxygen species mediate apoptosis, activate MMPs, and promote increased myofibroblast content through the MAPK and TLR pathways, resulting in adverse events after AMI [28]. Apoptosis triggers the inflammatory cascade in AMI.

The immune response is an important part of the inflammatory response and is mediated by immune cells. In this study, we conducted ssGSEA and quanTIseq immune infiltration analyses to evaluate infiltration of immune cells in AMI and with the role of the identified key genes in this process. The results showed that levels of neutrophils and macrophages were higher in the AMI group than those in the control group. Neutrophils and macrophages were positively associated with key genes in our study, which indicated that they may play an important role in the onset and progression of AMI. Macrophages, which develop from monocytes, play a central role in coronary heart disease, and are closely related to the inflammatory response, myocardial fibrosis, cell debris removal, and ventricular remodeling in AMI [29]. M0 macrophages differentiate into M1 and M2 macrophages under different conditions. Pro-inflammatory M1 macrophages release a large number of pro-inflammatory cytokines and chemokines to amplify the myocardial inflammatory cascade. Anti-inflammatory M2 macrophages inhibit myocardial inflammation [30]. The three key genes were significantly associated with macrophages. HCK, a key regulator of phagocytosis in macrophages, was closely related to M2 macrophages. Our findings showed that the identified key genes were important in immune regulation in AMI. Moreover, neutrophils were the primary infiltrating immune cells in AMI, and correlated with the identified key genes in our study. The role and related mechanism of neutrophils were important in the occurrence and development in AMI.

Recent studies have shown an important relationship between neutrophils and tumors. Neutrophils are markers of acute inflammation, coordinate the activation and regulation of the adaptive immune response in chronic inflammation. Neutrophils are also a significant component of the tumor microenvironment. Studies have shown that neutrophils play a key role in the onset and progression of cancer. Tumor infiltrating neutrophils can maintain tumor growth through different mechanisms, including inhibition of T cell activation, promotion of genetic instability, tumor cell proliferation, angiogenesis, and metastasis [31].

Studies have shown that neutrophils that infiltrate into tumors can release matrix metalloproteinase-9 (MMP-9), resulting in angiogenesis and proliferation of tumor cells. Neutrophils also inhibit natural killer (NK) cell function. Some studies have shown that transferrin secreted by neutrophils binds with receptors expressed on breast cancer cells to provide iron to accelerate proliferation of tumor cells. Neutrophils are also the key source of angiogenic growth factors and chemokines (such as VEGF, MMP-9 and FGF-2), and prokineticin-2 (PROK2), which can trigger chronic angiogenesis and promote tumor progression. In addition, mobilization of neutrophils or chemokines and their receptors promote tumor metastasis [13].

In this study, we conducted pan-cancer analysis of key genes to characterize the association between AMI and tumors. Using TCGA database, we found that three key genes showed abnormally high expression in most cancers, which indicated they could participate in the biological behavior of tumors. Prognostic analysis showed that high expression of key genes could mediate poor prognosis of cancers.

Interestingly, studies have shown that there may be a relationship between coronary heart disease and tumors [11]. Heart disease is one of the main non tumor causes of death in patients with cancer. Anthracycline drugs used to treat many tumors may promote myocardial injury and induce coronary heart disease. In contrast, recent epidemiological and prospective cohort studies indicated that patients with heart disease were more likely to develop tumors. Another large retrospective study of more than 5,000 Japanese patients with heart disease concluded that the incidence of cancer was four times higher in the heart disease group than that of the control group [32].

Therefore, we conducted pan cancer analysis using our identified key genes (lipid metabolism and AMI related) to understand the potential association of these key genes with AMI and tumors. In our study, the key genes were highly expressed in most tumors and were associated with poor prognosis. These key genes may serve as a bridge between AMI and tumor, and may be potential therapeutic targets.

## Supplementary Materials

Supplementary Figures

Supplementary Table 1
