# Supplementary material for: Identification and validation of lipid metabolism-related key genes as novel biomarkers in acute myocardial infarction and pan-cancer analysis
Source: Aging (Albany NY). 2024 May 23;16(10):9127–46. doi: 10.18632/aging.205860 (PMC11164520; doi:10.18632/aging.205860)
Supplement: Supplementary Table 1 [file aging-16-205860-s002.doc]

**Supplementary Table 1. Top 20 enrichment terms of key genes by GO-BP, KEGG and Hallmark.**

| Term | ES | NES | pvalue | FDR | FWER |
| --- | --- | --- | --- | --- | --- |
| MONOCYTE_DIFFERENTIATION | 0.6123 | 2.1536 | 0 | 0.0004 | 0.025 |
| REGULATION_OF_PROTEIN_PHOSPHORYLATION | 0.4423 | 2.1543 | 0 | 0.0004 | 0.025 |
| NEGATIVE_REGULATION_OF_LEUKOCYTE_CELL_CELL_ADHESION | 0.5768 | 2.155 | 0 | 0.0004 | 0.025 |
| CELL_CHEMOTAXIS | 0.5838 | 2.1555 | 0 | 0.0004 | 0.025 |
| REGULATION_OF_LEUKOCYTE_PROLIFERATION | 0.5715 | 2.1568 | 0 | 0.0004 | 0.024 |
| REGULATION_OF_PROTEIN_SERINE_THREONINE_KINASE_ACTIVITY | 0.5033 | 2.1571 | 0 | 0.0004 | 0.023 |
| POSITIVE_REGULATION_OF_PROTEIN_MODIFICATION_PROCESS | 0.4456 | 2.1573 | 0 | 0.0004 | 0.023 |
| MACROPHAGE_ACTIVATION | 0.7567 | 2.1573 | 0 | 0.0004 | 0.023 |
| SUPEROXIDE_METABOLIC_PROCESS | 0.6647 | 2.1615 | 0 | 0.0004 | 0.023 |
| T_CELL_DIFFERENTIATION_INVOLVED_IN_IMMUNE_RESPONSE | 0.6423 | 2.1627 | 0 | 0.0004 | 0.022 |
| IRON_ION_TRANSPORT | 0.5757 | 2.1633 | 0 | 0.0004 | 0.022 |
| RESPONSE_TO_LIPID | 0.4759 | 2.1634 | 0 | 0.0004 | 0.022 |
| EXOCYTOSIS | 0.6279 | 2.1641 | 0 | 0.0004 | 0.022 |
| POSITIVE_REGULATION_OF_SMOOTH_MUSCLE_CELL_PROLIFERATION | 0.6554 | 2.1666 | 0 | 0.0004 | 0.021 |
| RECEPTOR_METABOLIC_PROCESS | 0.5435 | 2.1668 | 0 | 0.0004 | 0.021 |
| REGULATION_OF_INFLAMMATORY_RESPONSE | 0.5553 | 2.1678 | 0 | 0.0004 | 0.021 |
| POSITIVE_REGULATION_OF_LEUKOCYTE_PROLIFERATION | 0.6036 | 2.169 | 0 | 0.0004 | 0.021 |
| REGULATION_OF_CELL_ADHESION | 0.474 | 2.17 | 0 | 0.0004 | 0.021 |
| REGULATION_OF_ORGANIC_ACID_TRANSPORT | 0.6432 | 2.17 | 0 | 0.0004 | 0.021 |
| MYELOID_DENDRITIC_CELL_DIFFERENTIATIONMYELOID_DENDRITIC_CELL_DIFFERENTIATION | 0.6892 | 2.1709 | 0 | 0.0004 | 0.02 |

**GSEA-TLR2-GO-BP**

|  | ES | NES | pvalue | FDR | FWER |
| --- | --- | --- | --- | --- | --- |
| RESPONSE_TO_MANGANESE_ION | 0.805 | 2.2967 | 0 | 0 | 0 |
| REGULATION_OF_PROTEIN_KINASE_ACTIVITY | 0.444 | 2.0278 | 0 | 0.0035 | 0.169 |
| REGULATION_OF_PRODUCTION_OF_MOLECULAR_MEDIATOR_OF_IMMUNE_RESPONSE | 0.591 | 2.0287 | 0 | 0.0035 | 0.167 |
| POSITIVE_REGULATION_OF_CHEMOKINE_PRODUCTION | 0.7174 | 2.0376 | 0 | 0.0035 | 0.148 |
| NEGATIVE_REGULATION_OF_DEFENSE_RESPONSE | 0.5131 | 2.0379 | 0 | 0.0035 | 0.147 |
| POSITIVE_REGULATION_OF_PRODUCTION_OF_MOLECULAR_MEDIATOR_OF_IMMUNE_RESPONSE | 0.601 | 2.0385 | 0 | 0.0035 | 0.146 |
| REGULATION_OF_INFLAMMATORY_RESPONSE | 0.5238 | 2.0392 | 0 | 0.0035 | 0.144 |
| POSITIVE_REGULATION_OF_LEUKOCYTE_MIGRATION | 0.5587 | 2.0395 | 0 | 0.0035 | 0.142 |
| REGULATION_OF_PROTEIN_SERINE_THREONINE_KINASE_ACTIVITY | 0.4759 | 2.0403 | 0 | 0.0035 | 0.14 |
| REGULATION_OF_IMMUNE_SYSTEM_PROCESS | 0.5126 | 2.0424 | 0 | 0.0035 | 0.136 |
| LEUKOCYTE_DIFFERENTIATION | 0.5038 | 2.0782 | 0 | 0.0035 | 0.088 |
| RECEPTOR_INTERNALIZATION | 0.5515 | 2.0795 | 0 | 0.0035 | 0.085 |
| POSITIVE_REGULATION_OF_CELL_ACTIVATION | 0.6097 | 2.0798 | 0 | 0.0035 | 0.084 |
| POSITIVE_REGULATION_OF_DEFENSE_RESPONSE | 0.5701 | 2.0269 | 0 | 0.0036 | 0.172 |
| NEGATIVE_REGULATION_OF_IMMUNE_RESPONSE | 0.5583 | 2.0287 | 0 | 0.0036 | 0.167 |
| REGULATION_OF_APOPTOTIC_SIGNALING_PATHWAY | 0.524 | 2.0287 | 0 | 0.0036 | 0.167 |
| CYTOKINE_MEDIATED_SIGNALING_PATHWAY | 0.5262 | 2.0313 | 0 | 0.0036 | 0.159 |
| REGULATION_OF_LYMPHOCYTE_ACTIVATION | 0.554 | 2.0317 | 0 | 0.0036 | 0.159 |
| LIPOPOLYSACCHARIDE_MEDIATED_SIGNALING_PATHWAY | 0.7991 | 2.0323 | 0 | 0.0036 | 0.158 |
| POSITIVE_REGULATION_OF_REACTIVE_OXYGEN_SPECIES_METABOLIC_PROCESS | 0.6605 | 2.0326 | 0 | 0.0036 | 0.158 |

**GSEA-S100A9-GO-BP**

| Term | ES | NES | pvalue | FDR | FWER |
| --- | --- | --- | --- | --- | --- |
| DEFENSE_RESPONSE | 0.431 | 1.8452 | 0.0019 | 0.0339 | 0.711 |
| POSITIVE_REGULATION_OF_REACTIVE_OXYGEN_SPECIES_BIOSYNTHETIC_PROCESS | 0.6677 | 1.8422 | 0.0039 | 0.034 | 0.719 |
| POSITIVE_REGULATION_OF_REACTIVE_OXYGEN_SPECIES_METABOLIC_PROCESS | 0.6137 | 1.9259 | 0.0019 | 0.034 | 0.471 |
| GERMINAL_CENTER_FORMATION | 0.8185 | 1.8417 | 0 | 0.0341 | 0.725 |
| DEFENSE_RESPONSE_TO_OTHER_ORGANISM | 0.4479 | 1.8427 | 0.002 | 0.0341 | 0.719 |
| POSITIVE_REGULATION_OF_LEUKOCYTE_MEDIATED_IMMUNITY | 0.5621 | 1.8453 | 0 | 0.0341 | 0.711 |
| LIPID_STORAGE | 0.4871 | 1.9162 | 0.0038 | 0.0341 | 0.514 |
| REACTIVE_OXYGEN_SPECIES_METABOLIC_PROCESS | 0.4802 | 1.8457 | 0.0041 | 0.0342 | 0.71 |
| POSITIVE_REGULATION_OF_DNA_BINDING_TRANSCRIPTION_FACTOR_ACTIVITYDNA | 0.4544 | 1.8428 | 0 | 0.0343 | 0.719 |
| REGULATION_OF_GRANULOCYTE_CHEMOTAXIS | 0.5657 | 1.8459 | 0.0078 | 0.0343 | 0.71 |
| LYMPHOCYTE_ACTIVATION | 0.4934 | 1.8466 | 0 | 0.0343 | 0.71 |
| POSITIVE_REGULATION_OF_INTERLEUKIN_8_PRODUCTION | 0.6738 | 1.8958 | 0.0038 | 0.0343 | 0.587 |
| NEGATIVE_REGULATION_OF_LYMPHOCYTE_ACTIVATION | 0.5157 | 1.8965 | 0 | 0.0343 | 0.581 |
| REGULATION_OF_PRODUCTION_OF_MOLECULAR_MEDIATOR_OF_IMMUNE_RESPONSE | 0.5552 | 1.917 | 0 | 0.0343 | 0.511 |
| POSITIVE_REGULATION_OF_CELL_CELL_ADHESION | 0.4916 | 1.8431 | 0 | 0.0344 | 0.715 |
| REGULATION_OF_SYNCYTIUM_FORMATION_BY_PLASMA_MEMBRANE_FUSION | 0.626 | 1.8436 | 0.008 | 0.0344 | 0.714 |
| POSITIVE_REGULATION_OF_REGULATED_SECRETORY_PATHWAY | 0.5142 | 1.8529 | 0.0039 | 0.0344 | 0.701 |
| REGULATION_OF_LIPID_STORAGE | 0.4926 | 1.8403 | 0.0096 | 0.0345 | 0.733 |
| LYMPHOCYTE_MEDIATED_IMMUNITY | 0.5158 | 1.8533 | 0 | 0.0345 | 0.701 |
| REGULATION_OF_CELL_CELL_ADHESION | 0.4444 | 1.8466 | 0 | 0.0346 | 0.71 |

**GSEA-HCK-GO-BP**

| Term | ES | NES | pvalue | FDR | FWER |
| --- | --- | --- | --- | --- | --- |
| TOLL_LIKE_RECEPTOR_SIGNALING_PATHWAY | 0.6627 | 2.158 | 0 | 0 | 0 |
| EPITHELIAL_CELL_SIGNALING_IN_HELICOBACTER_PYLORI_INFECTION | 0.6774 | 2.1669 | 0 | 0 | 0 |
| HEMATOPOIETIC_CELL_LINEAGE | 0.6553 | 2.1703 | 0 | 0 | 0 |
| CHEMOKINE_SIGNALING_PATHWAY | 0.5843 | 2.2057 | 0 | 0 | 0 |
| MAPK_SIGNALING_PATHWAY | 0.5021 | 2.3449 | 0 | 0 | 0 |
| NOD_LIKE_RECEPTOR_SIGNALING_PATHWAY | 0.7023 | 2.0596 | 0 | 0.0001 | 0.001 |
| FC_EPSILON_RI_SIGNALING_PATHWAY | 0.5897 | 2.075 | 0 | 0.0001 | 0.001 |
| NATURAL_KILLER_CELL_MEDIATED_CYTOTOXICITY | 0.6089 | 2.0852 | 0 | 0.0001 | 0.001 |
| LEUKOCYTE_TRANSENDOTHELIAL_MIGRATION | 0.5126 | 2.0974 | 0 | 0.0001 | 0.001 |
| PATHWAYS_IN_CANCER | 0.4261 | 2.1005 | 0 | 0.0002 | 0.001 |
| VEGF_SIGNALING_PATHWAY | 0.5295 | 2.1178 | 0 | 0.0002 | 0.001 |
| LEISHMANIA_INFECTION | 0.8072 | 2.048 | 0 | 0.0003 | 0.003 |
| TYPE_I_DIABETES_MELLITUS | 0.6857 | 2.0308 | 0 | 0.0004 | 0.004 |
| FC_GAMMA_R_MEDIATED_PHAGOCYTOSIS | 0.6358 | 2.0056 | 0 | 0.0007 | 0.007 |
| SYSTEMIC_LUPUS_ERYTHEMATOSUS | 0.6224 | 1.9842 | 0 | 0.0013 | 0.013 |
| ADIPOCYTOKINE_SIGNALING_PATHWAY | 0.5004 | 1.9527 | 0 | 0.0019 | 0.022 |
| ASTHMA | 0.7077 | 1.9477 | 0 | 0.0022 | 0.027 |
| ALLOGRAFT_REJECTION | 0.7154 | 1.9314 | 0 | 0.0026 | 0.035 |
| GRAFT_VERSUS_HOST_DISEASE | 0.7946 | 1.9356 | 0 | 0.0026 | 0.033 |
| ERBB_SIGNALING_PATHWAY | 0.4698 | 1.9115 | 0 | 0.0027 | 0.038 |

GSEA-TLR2-KEGG

| Term | ES | NES | pvalue | FDR | FWER |
| --- | --- | --- | --- | --- | --- |
| NOD_LIKE_RECEPTOR_SIGNALING_PATHWAY | 0.7075 | 2.0875 | 0 | 0.0034 | 0.004 |
| FC_GAMMA_R_MEDIATED_PHAGOCYTOSIS/FCγR | 0.6064 | 1.9511 | 0.002 | 0.0035 | 0.029 |
| TOLL_LIKE_RECEPTOR_SIGNALING_PATHWAY | 0.6274 | 2.0383 | 0 | 0.0035 | 0.007 |
| MAPK_SIGNALING_PATHWAY | 0.4503 | 2.1233 | 0 | 0.0036 | 0.003 |
| LEUKOCYTE_TRANSENDOTHELIAL_MIGRATION | 0.4811 | 1.9543 | 0.0021 | 0.0037 | 0.028 |
| LEISHMANIA_INFECTION | 0.7714 | 1.9878 | 0 | 0.0037 | 0.018 |
| RENAL_CELL_CARCINOMA | 0.55 | 1.9717 | 0 | 0.0039 | 0.023 |
| HEMATOPOIETIC_CELL_LINEAGE | 0.5833 | 1.9374 | 0.0019 | 0.004 | 0.036 |
| PATHWAYS_IN_CANCER | 0.3944 | 1.9576 | 0 | 0.004 | 0.028 |
| FC_EPSILON_RI_SIGNALING_PATHWAY | 0.5499 | 1.9644 | 0 | 0.004 | 0.025 |
| PATHOGENIC_ESCHERICHIA_COLI_INFECTION | 0.6693 | 1.9881 | 0 | 0.0042 | 0.018 |
| NATURAL_KILLER_CELL_MEDIATED_CYTOTOXICITY | 0.5638 | 1.9736 | 0 | 0.0043 | 0.023 |
| EPITHELIAL_CELL_SIGNALING_IN_HELICOBACTER_PYLORI_INFECTION | 0.6303 | 1.9977 | 0 | 0.0044 | 0.016 |
| PROSTATE_CANCER | 0.49 | 1.9177 | 0 | 0.0045 | 0.044 |
| NOTCH_SIGNALING_PATHWAY | 0.5203 | 1.9007 | 0.004 | 0.0052 | 0.054 |
| BLADDER_CANCER | 0.598 | 1.9991 | 0 | 0.0052 | 0.016 |
| SYSTEMIC_LUPUS_ERYTHEMATOSUS | 0.5893 | 1.878 | 0.004 | 0.0064 | 0.068 |
| VIRAL_MYOCARDITIS | 0.527 | 1.8712 | 0.002 | 0.0067 | 0.074 |
| TYPE_I_DIABETES_MELLITUS | 0.6197 | 1.8476 | 0 | 0.0069 | 0.085 |
| GRAFT_VERSUS_HOST_DISEASE | 0.7691 | 1.8595 | 0 | 0.0069 | 0.08 |

**GSEA-S100A9-KEGG**

| Term | ES | NES | pvalue | | FDR | FWER |
| --- | --- | --- | --- | --- | --- | --- |
| CHEMOKINE_SIGNALING_PATHWAY | 0.5164 | 1.9306 | | 0 | 0.0112 | 0.03 |
| EPITHELIAL_CELL_SIGNALING_IN_HELICOBACTER_PYLORI_INFECTION | 0.605 | 1.9428 | | 0 | 0.0135 | 0.028 |
| LEISHMANIA_INFECTION | 0.7634 | 1.9618 | | 0 | 0.0159 | 0.023 |
| MAPK_SIGNALING_PATHWAY | 0.3936 | 1.8257 | | 0 | 0.0185 | 0.105 |
| NOD_LIKE_RECEPTOR_SIGNALING_PATHWAY | 0.6168 | 1.8003 | | 0.004 | 0.0188 | 0.135 |
| LYSOSOME | 0.5674 | 1.8057 | | 0 | 0.019 | 0.125 |
| B_CELL_RECEPTOR_SIGNALING_PATHWAY | 0.6737 | 1.8117 | | 0 | 0.0195 | 0.119 |
| FC_EPSILON_RI_SIGNALING_PATHWAY | 0.5143 | 1.8298 | | 0 | 0.0198 | 0.102 |
| FC_GAMMA_R_MEDIATED_PHAGOCYTOSIS | 0.5705 | 1.836 | | 0.0041 | 0.0198 | 0.092 |
| HEMATOPOIETIC_CELL_LINEAGE | 0.572 | 1.8365 | | 0.0081 | 0.0226 | 0.092 |
| APOPTOSIS | 0.5864 | 1.7747 | | 0 | 0.0238 | 0.165 |
| VEGF_SIGNALING_PATHWAY | 0.4647 | 1.865 | | 0.004 | 0.0246 | 0.073 |
| NOTCH_SIGNALING_PATHWAY | 0.5442 | 1.9814 | | 0.002 | 0.0261 | 0.019 |
| TOLL_LIKE_RECEPTOR_SIGNALING_PATHWAY | 0.5612 | 1.8378 | | 0.0081 | 0.0263 | 0.092 |
| RENAL_CELL_CARCINOMA | 0.4863 | 1.7582 | | 0.0085 | 0.0268 | 0.184 |
| LEUKOCYTE_TRANSENDOTHELIAL_MIGRATION | 0.4275 | 1.7433 | | 0.0142 | 0.03 | 0.212 |
| AMYOTROPHIC_LATERAL_SCLEROSIS_ALS | 0.4711 | 1.7272 | | 0.006 | 0.0342 | 0.242 |
| NON_SMALL_CELL_LUNG_CANCER | 0.4871 | 1.7017 | | 0.0041 | 0.0359 | 0.291 |
| CHRONIC_MYELOID_LEUKEMIA | 0.5117 | 1.7105 | | 0.002 | 0.0359 | 0.271 |
| T_CELL_RECEPTOR_SIGNALING_PATHWAY | 0.578 | 1.6932 | | 0 | 0.0362 | 0.317 |

**GSEA-HCK-KEGG**

| Term | ES | NES | pvalue | FDR | FWER |
| --- | --- | --- | --- | --- | --- |
| INFLAMMATORY_RESPONSE | 0.7108 | 2.2407 | 0 | 0 | 0 |
| IL6_JAK_STAT3_SIGNALING | 0.7272 | 2.1444 | 0 | 0.0005 | 0.001 |
| COMPLEMENT | 0.6395 | 2.0872 | 0 | 0.0006 | 0.002 |
| KRAS_SIGNALING_UP | 0.5913 | 2.0759 | 0 | 0.0007 | 0.003 |
| UV_RESPONSE_UP | 0.5473 | 2.0892 | 0 | 0.0008 | 0.002 |
| TNFA_SIGNALING_VIA_NFKB | 0.8123 | 2.0129 | 0 | 0.0012 | 0.007 |
| IL2_STAT5_SIGNALING | 0.5498 | 2.0109 | 0 | 0.0014 | 0.008 |
| APOPTOSIS | 0.6432 | 2.0138 | 0 | 0.0014 | 0.007 |
| P53_PATHWAY | 0.5575 | 1.9041 | 0 | 0.0024 | 0.013 |
| XENOBIOTIC_METABOLISM | 0.4367 | 1.9082 | 0 | 0.0025 | 0.013 |
| ALLOGRAFT_REJECTION | 0.6251 | 1.8798 | 0 | 0.0031 | 0.022 |
| HYPOXIA | 0.515 | 1.8805 | 0.0019 | 0.0033 | 0.021 |
| COAGULATION | 0.5462 | 1.831 | 0.0038 | 0.0045 | 0.033 |
| TGF_BETA_SIGNALING | 0.6178 | 1.8134 | 0 | 0.0049 | 0.037 |
| BILE_ACID_METABOLISM | 0.418 | 1.7885 | 0.002 | 0.0063 | 0.047 |
| HEME_METABOLISM | 0.4878 | 1.7656 | 0 | 0.0075 | 0.055 |
| APICAL_JUNCTION | 0.3705 | 1.7265 | 0.0019 | 0.0109 | 0.077 |
| ESTROGEN_RESPONSE_LATE | 0.4217 | 1.7187 | 0.0075 | 0.0111 | 0.081 |
| CHOLESTEROL_HOMEOSTASIS | 0.493 | 1.6976 | 0.0134 | 0.0135 | 0.097 |
| MITOTIC_SPINDLE | 0.4571 | 1.6666 | 0.002 | 0.0169 | 0.119 |

**GSEA-TLR2-Hallmark**

| Term | ES | NES | pvalue | FDR | FWER |
| --- | --- | --- | --- | --- | --- |
| UV_RESPONSE_UP | 0.533 | 2.0093 | 0 | 0.0004 | 0.002 |
| APOPTOSIS | 0.6587 | 2.0417 | 0 | 0.0004 | 0.002 |
| INFLAMMATORY_RESPONSE | 0.6599 | 2.048 | 0 | 0.0006 | 0.002 |
| KRAS_SIGNALING_UP | 0.5578 | 1.9864 | 0 | 0.0007 | 0.004 |
| TNFA_SIGNALING_VIA_NFKB | 0.8219 | 2.0501 | 0 | 0.0007 | 0.002 |
| IL2_STAT5_SIGNALING | 0.5348 | 1.9581 | 0 | 0.0009 | 0.006 |
| HYPOXIA | 0.5366 | 1.9451 | 0.002 | 0.0011 | 0.009 |
| IL6_JAK_STAT3_SIGNALING | 0.7035 | 2.054 | 0 | 0.0011 | 0.002 |
| CHOLESTEROL_HOMEOSTASIS | 0.5417 | 1.9106 | 0.004 | 0.0014 | 0.011 |
| ANDROGEN_RESPONSE | 0.5262 | 1.917 | 0.002 | 0.0015 | 0.011 |
| COMPLEMENT | 0.6371 | 2.0807 | 0 | 0.0022 | 0.002 |
| ALLOGRAFT_REJECTION | 0.6138 | 1.8647 | 0 | 0.0033 | 0.02 |
| P53_PATHWAY | 0.5459 | 1.8498 | 0 | 0.0035 | 0.026 |
| HEME_METABOLISM | 0.511 | 1.8584 | 0.002 | 0.0035 | 0.023 |
| TGF_BETA_SIGNALING | 0.627 | 1.8395 | 0.0041 | 0.0036 | 0.027 |
| INTERFERON_GAMMA_RESPONSE | 0.6586 | 1.811 | 0 | 0.0047 | 0.035 |
| MITOTIC_SPINDLE | 0.4821 | 1.7871 | 0.002 | 0.0058 | 0.045 |
| PI3K_AKT_MTOR_SIGNALING | 0.5218 | 1.7246 | 0 | 0.0105 | 0.076 |
| MTORC1_SIGNALING | 0.5896 | 1.7073 | 0.0081 | 0.0108 | 0.086 |
| COAGULATION | 0.5039 | 1.712 | 0.0079 | 0.0109 | 0.082 |

**GSEA-S100A9-Hallmark**

| Term | ES | NES | pvalue | FDR | FWER |
| --- | --- | --- | --- | --- | --- |
| IL6_JAK_STAT3_SIGNALING | 0.7034 | 2.1068 | 0 | 0.001 | 0.001 |
| INFLAMMATORY_RESPONSE | 0.6273 | 2.0002 | 0 | 0.0011 | 0.002 |
| TNFA_SIGNALING_VIA_NFKB | 0.7613 | 1.941 | 0 | 0.0045 | 0.01 |
| COMPLEMENT | 0.5607 | 1.8656 | 0 | 0.0069 | 0.019 |
| APOPTOSIS | 0.5871 | 1.8751 | 0.0019 | 0.0069 | 0.016 |
| IL2_STAT5_SIGNALING | 0.4913 | 1.7855 | 0.002 | 0.0169 | 0.047 |
| ALLOGRAFT_REJECTION | 0.5899 | 1.759 | 0 | 0.0182 | 0.053 |
| P53_PATHWAY | 0.5059 | 1.7198 | 0.002 | 0.0256 | 0.081 |
| HYPOXIA | 0.4638 | 1.6867 | 0.0163 | 0.0321 | 0.1 |
| TGF_BETA_SIGNALING | 0.567 | 1.6515 | 0.0144 | 0.0331 | 0.125 |
| INTERFERON_GAMMA_RESPONSE | 0.6108 | 1.6335 | 0.01 | 0.0333 | 0.141 |
| INTERFERON_ALPHA_RESPONSE | 0.6642 | 1.6384 | 0.004 | 0.0344 | 0.136 |
| UV_RESPONSE_UP | 0.4388 | 1.6516 | 0.0162 | 0.0361 | 0.125 |
| KRAS_SIGNALING_UP | 0.4682 | 1.6606 | 0.0261 | 0.0366 | 0.119 |
| HEME_METABOLISM | 0.4237 | 1.5301 | 0.0477 | 0.0834 | 0.292 |
| PI3K_AKT_MTOR_SIGNALING | 0.4744 | 1.511 | 0.0202 | 0.0922 | 0.323 |
| REACTIVE_OXYGEN_SPECIES_PATHWAY | 0.5643 | 1.467 | 0.0524 | 0.1133 | 0.38 |
| ADIPOGENESIS | 0.4238 | 1.4743 | 0.0223 | 0.114 | 0.371 |
| PROTEIN_SECRETION | 0.53 | 1.4522 | 0.0716 | 0.1207 | 0.41 |
| MITOTIC_SPINDLE | 0.3854 | 1.4043 | 0.0652 | 0.1517 | 0.479 |

**GSEA-HCK-Hallmark**
